# Supplementary material for: Roles of the ClC chloride channel CLH-1 in food-associated salt chemotaxis behavior of C. elegans
Source: eLife. 2021 Jan 25;10:e55701. doi: 10.7554/eLife.55701 (PMC7834019; doi:10.7554/eLife.55701)
Supplement: Supplementary file 1. [file elife-55701-supp1.docx]

| **Strains used in this study** | | |
| --- | --- | --- |
| **Strain** | **Genotype** | **Source** |
| N2 | *C. elegans* wild type. | CGC |
| JN572 | *clh-1(pe572) II*. | This study |
| JN577 | *clh-1(pe577) II*. | This study |
| JN1638 | *clh-1(tm1243) II.* | NBRP ^(a)^ |
| JN1639 | *clh-1(ok658) II*. | CGC ^(a)^ |
| JN2285 | *clh-1(qa901) II* | CGC ^(a)^ |
| PD4788 | *mIs13 I.* | From M. Edgley  (Rea et al., 2005) |
| JN2376 | *Ex[myo-3p::Venus].* | This study |
| JN1644 | *clh-1(pe572) II; Ex[myo-3p::Venus].* | This study |
| JN1645 | *clh-1(pe577) II; Ex[myo-3p::Venus].* | This study |
| - | *Ex[clh-1(wt)gDNA; myo-3p::venus].* | This study |
| - | *clh-1(pe572) II; Ex[clh-1(wt)gDNA; myo-3p::venus].* | This study |
| - | *clh-1(pe577) II; Ex[clh-1(wt)gDNA; myo-3p::venus].* | This study |
| JN2230 | *mIs13 I; clh-1(tm1243) II.* | This study |
| CB1072 | *unc-29(e1072) I.* | CGC |
| JN2207 | *clh-1(tm1243) II; Ex[myo-3p::venus].* | This study |
| JN2263 | *peEx2263[clh-1(pe572)gDNA; myo-3p::venus].* | This study |
| JN2265 | *clh-1(tm1243) II; peEx2263[clh-1(pe572)gDNA; myo-3p::venus].* | This study |
| JN2267 | *peEx2267[clh-1(pe577)gDNA; myo-3p::venus].* | This study |
| JN2269 | *clh-1(tm1243) II; peEx2267[clh-1(pe577)gDNA; myo-3p::venus].* | This study |
| JN2231 | *clh-2(ok636) II*. | CGC ^(a)^ |
| JN2260 | *clh-3(ok763) II*. | CGC ^(a)^ |
| JN2286 | *clh-4(ok1162) II*. | CGC ^(a)^ |
| JN2261 | *clh-5(tm6008) II*. | NBRP ^(a)^ |
| JN2262 | *clh-6(tm617) V.* | NBRP ^(a)^ |
| JN2371 | *clh-2(ok636) clh-1(pe572) II.* | This study |
| JN2372 | *clh-3(ok763) clh-2(ok636) clh-1(pe572) II.* | This study |
| JN2393 | *clh-1(pe572) II; clh-4(ok1162) X.* | This study |
| JN2396 | *clh-5(tm6008) clh-1(pe572) II.* | This study |
| JN2395 | *clh-1(pe572) II; clh-6(tm617) V.* | This study |
| JN2250 | *clh-2(ok636) clh-1(tm1243) II.* | This study |
| JN2288 | *clh-3(ok763) clh-2(ok636) clh-1(tm1243) II.* | This study |
| JN2289 | *clh-2(ok636) clh-1(tm1243) II; clh-4(ok1162) X.* | This study |
| JN2290 | *clh-5(tm6008) clh-2(ok636) clh-1(tm1243) II.* | This study |
| JN2291 | *clh-2(ok636) clh-1(tm1243) II; clh-6(tm617) V.* | This study |
| JN2325 | *clh-3(ok763) clh-2(ok636) clh-1(tm1243) II; clh-4(ok1162) X.* | This study |
| JN2326 | *clh-3(ok763) clh-5(tm6008) clh-2(ok636) clh-1(tm1243) II.* | This study |
| JN2604 | *clh-3(ok763) clh-5(tm6008) clh-2(ok636) clh-1(pe572) II; clh-6(tm617) V; clh-4(ok1162) X.* | This study |
| JN2600 | *clh-3(ok763) clh-5(tm6008) clh-2(ok636) clh-1(tm1243) II; clh-6(tm617) V; clh-4(ok1162) X.* | This study |
| JN2249 | *Is[rimb-1p::nls4::mCherry; eat-4p::nls4::tagRFP; lin-44p::GFP]; peEx2249[clh-1p::SL2::nls4::mTFP; lin-44p::mCherry].* | This study |
| JN2241 | *clh-1(pe572) II; peEx2241[rimb-1p::clh-1(wt)cDNA; myo-3p::Venus].* | This study |
| JN2243 | *clh-1(pe572) II; peEx2243[gcy-5p::clh-1(wt)cDNA; myo-3p::Venus].* | This study |
| JN2244 | *clh-1(pe572) II; peEx2244[gcy-7p::clh-1(wt)cDNA; myo-3p::Venus].* | This study |
| JN2245 | *clh-1(pe572) II; peEx2245[gcy-5p::clh-1(wt)cDNA; gcy-7p::clh-1(wt)cDNA; myo-3p::Venus].* | This study |
| JN2242 | *clh-1(pe572) II; peEx2242[dyf-11p::clh-1(wt)cDNA; myo-3p::Venus].* | This study |
| JN2247 | *clh-1(pe572) II; peEx2247[vap-1p::clh-1(wt)cDNA; myo-3::Venus].* | This study |
| JN2246 | *clh-1(pe572) II; peEx2246[gcy-5p::clh-1(wt)cDNA; vap-1p::clh-1(wt)cDNA; myo-3p::Venus].* | This study |
| JN2229 | *clh-1(pe577) II; peEx2229[gcy-5p::clh-1(wt)cDNA; myo-3p::Venus].* | This study |
| OH3192 | *ntIs1[lin-15(+); gcy-5p::GFP] V.* | CGC  (Tomioka et al., 2006) |
| JN2377 | *clh-1(pe572) II; ntIs1[lin-15(+); gcy-5p::GFP] V.* | This study |
| JN2378 | *clh-1(pe577) II; ntIs1[lin-15(+); gcy-5p::GFP] V.* | This study |
| JN2379 | *clh-1(tm1243) II; ntIs1[lin-15(+); gcy-5p::GFP] V.* | This study |
| JN2215 | *ntIs1[lin-15(+); gcy-5p::GFP] V; peEx2215[vap-1::mCherry].* | This study |
| JN2217 | *clh-1(pe577) II; ntIs1[lin-15(+); gcy-5p::GFP] V ; peEx2215[vap-1p::mCherry].* | This study |
| JN2218 | *clh-1(tm1243) II; ntIs1[lin-15(+); gcy-5p::GFP] V ; peEx2215[vap-1p::mCherry].* | This study |
| JN2255 | *clh-1(pe572) II; peEx2255[lin-44p::GFP].* | This study |
| JN2248 | *clh-1(pe572) II; peEx2248[gcy-5p::clh-1(wt)cDNA::mTFP; lin-44p::GFP].* | This study |
| JN2222 | *peEx2222[gcy-5p::clh-1(wt)cDNA::mTFP; lin-44p::GFP].* | This study |
| JN2256 | *clh-1(pe572) II; peEx2223[gcy-5p::clh-1(pe572)cDNA::mTFP; lin-44p::GFP].* | This study |
| JN2321 | *peEx2321[gcy-5p::CeSuperclomeleon; lin-44p::mCherry].* | This study |
| JN2322 | *clh-1(pe572) II; peEx2321[gcy-5p::CeSuperclomeleon; lin-44p::mCherry].* | This study |
| JN2323 | *clh-1(pe577) II; peEx2321[gcy-5p::CeSuperclomeleon; lin-44p::mCherry].* | This study |
| JN2324 | *clh-1(tm1243) II; peEx2321[gcy-5p::CeSuperclomeleon; lin-44p::mCherry].* | This study |
| JN2382 | *clh-1(pe572); peEx2352[gcy-5p::clh-1(wt)cDNA; lin-44p::GFP]; peEx2321[gcy-5p::CeSuperclomeleon; lin-44p::mCherry]* | This study |
| JN2102 | *Ex[gcy-5p::YC2.60; lin-44p::mCherry].* | (Jang et al., 2019) |
| JN2219 | *clh-1(pe572) II; Ex[gcy-5p::YC2.60; lin-44p::mCherry].* | This study |
| JN2220 | *clh-1(pe577) II; Ex[gcy-5p::YC2.60; lin-44p::mCherry].* | This study |
| JN2221 | *clh-1(tm1243) II; Ex[gcy-5p::YC2.60; lin-44p::mCherry].* | This study |
| JN2380 | *clh-1(pe572); peEx2352[gcy-5p::clh-1(wt)cDNA; lin-44p::GFP]; Ex[gcy-5p::YC2.60; lin-44p::mCherry].* | This study |
| JN2391 | *peEx2391[clh-1p::SL2::NLS4::mTFP; gcy-5p::mCherry; lin-44p::GFP]* | This study |
| JN2392 | *clh-1(pe572) II; peEx2392[clh-1p::SL2::NLS4::mTFP; gcy-5p::mCherry; lin-44p::GFP]* | This study |
| JN2605 | *clh-1(pe577) II; peEx2391[clh-1p::SL2::NLS4::mTFP; gcy-5p::mCherry; lin-44p::GFP]* | This study |
| JN3329 | *Ex[npr-9p::GCaMP6s; npr-9p::mCherry; lin-44p::GFP].* | This study |
| JN2345 | *clh-1(pe572); Ex[npr-9p::GCaMP6s; npr-9p::mCherry; lin-44p::GFP].* | This study |
| JN2383 | *clh-1(pe572); peEx2351[gcy-5p::clh-1(wt)cDNA; lin-44p::mCherry]; Ex[npr-9p::GC6s/mCherry, lin-44p::GFP]* | This study |
| a: These strains were outcrossed to N2 four to eight times in our lab before use. | | |
