# Supplementary material for: Roles of the ClC chloride channel CLH-1 in food-associated salt chemotaxis behavior of C. elegans
Source: eLife. 2021 Jan 25;10:e55701. doi: 10.7554/eLife.55701 (PMC7834019; doi:10.7554/eLife.55701)
Supplement: Supplementary file 2. [file elife-55701-supp2.docx]

| **Genotyping primers used in this study** | | |
| --- | --- | --- |
| **Primer name** | | **Primer Sequence 5’ → 3’** |
| *clh-1(pe572)* | F ^(a)^ | CTTGCACATTCTCGGCGCATAT |
| *clh-1(pe572)* | R ^(a)^ | GCAGGTAGTGGCAAAGAATG |
| *clh-1(pe577)* | F | TTGTCGAGGACTGGTTCATC |
| *clh-1(pe577)* | R | GACTCACAATCAGATACTGA |
| *clh-1(tm1243)* | F1 | TGGTGTGCTATGGATTTGGA |
| *clh-1(tm1243)* | F2 | TTTGTTGTCGAGGAATTGCC |
| *clh-1(tm1243)* | R | CGAAGAGAGCTCCGAGAAGA |
| *clh-1(ok658)* | F1 | CCTCAGCACCTCCCCAAATTA |
| *clh-1(ok658)* | F2 | CCGAAACTTCATGCTAGAGTC |
| *clh-1(ok658)* | R | AAAACCCCAAACCCAAAATC |
| *clh-1(qa901)* | F1 | CCTAGTAAAGAGGCAGGTACGTG |
| *clh-1(qa901)* | F2 | CCAACTGTACCCTCTGGAAGC |
| *clh-1(qa901)* | R | CGGCATAAAGTCCAGGGTAGATC |
| *clh-2(ok636)* | F1 | CTTCCTCGCCTTCACTGTGTTC |
| *clh-2(ok636)* | F2 | TGGTAAACCAGCAGGACAGTCG |
| *clh-2(ok636)* | R1 | GGCAATGGAGAGGAGATATTGAGAG |
| *clh-3(ok763)* | F1 | CCCATCTTCATCGCCACTTTC |
| *clh-3(ok763)* | F2 | CGTACTGAATGGGAGGATATGATG |
| *clh-3(ok763)* | R | CCCATCTTCATCGCCACTTTC |
| *clh-4(ok1162)* ^(b)^ | F | GTCAGCGCGTTCTACCAAACAAG |
| *clh-4(ok1162)* ^(b)^ | R1 | GCTCCGCAAACAAGATCATCTGAC |
| *clh-4(ok1162)* ^(b)^ | R2 | CAAGCCAAAGATCCTGAATGCTCC |
| *clh-5(tm6008)* | F1 | GGAGATCATTCTTCTGTGCATTGG |
| *clh-5(tm6008)* | F2 | CTTCTCTGGTCCTCTCGTAAC |
| *clh-5(tm6008)* | R | GCGTGATTAGCATGACTTCGTAG |
| *clh-6(tm617)* | F1 | GCTCCAAGACAGCCTCCAATTATTC |
| *clh-6(tm617)* | F2 | CCTATCCAAGCTATCATTACGGTCCAC |
| *clh-6(tm617)* | R | CTGGACTCGTCGCTGCATTTATTG |
| a: F signifies forward and R signifies reverse. | | |
| b: in *clh-4(ok1162)*, it has revealed by sequencing that 2.6 kb between exon7 and exon 18 were substituted to another 1.4 kb sequence in the allele, which mutation presumably destroy the function of CLH-4 protein. | | |
