# Supplementary material for: Roles of the ClC chloride channel CLH-1 in food-associated salt chemotaxis behavior of C. elegans
Source: eLife. 2021 Jan 25;10:e55701. doi: 10.7554/eLife.55701 (PMC7834019; doi:10.7554/eLife.55701)
Supplement: Supplementary file 3. [file elife-55701-supp3.docx]

| **Plasmid constructions used in this study** | |
| --- | --- |
| **Constructed Plasmid Name** | **Use** |
| pG-*clh-1p::SL2::NLS4::mTFP* | *clh-1* gene expression pattern annotation and *in vivo* quantification of *clh-1p* expression level in ASER |
| pG-*rimb-1p::clh-1(wt)cDNA* | Rescue experiment |
| pG-*gcy-5p::clh-1(wt)cDNA* | Rescue experiment |
| pG-*gcy-7p::clh-1(wt)cDNA* | Rescue experiment |
| pG-*dyf-11p::clh-1(wt)cDNA* | Rescue experiment |
| pG-*vap-1p::clh-1(wt)cDNA* | Rescue experiment |
| pG-*vap-1p::mCherry* | Morphology observation |
| pG-*gcy-5p::clh-1(wt)cDNA::mTFP* | Intracellular localization observation |
| pG-*gcy-5p::clh-1(pe572)cDNA::mTFP* | Intracellular localization observation |
| pCDNA3.1-*clh-1cDNA(pe572)* | *In vivo* electrophysiology experiment in *X. Laevis* oocyte |
| pCDNA3.1-*clh-1cDNA(pe577)* | *In vivo* electrophysiology experiment in *X. Laevis* oocyte |
| pCDNA3.1-*clh-1cDNA(wt)* | *In vivo* electrophysiology experiment in *X. Laevis* oocyte |
| pG-*gcy-5p::Superclomeleon* | *In vivo* chloride imaging of ASER |
| pG-*gcy-5p::mCherry* | *In vivo* quantification of *clh-1p* expression level in ASER |
| pG-*npr-9p::GCaMP6s* | *In vivo* calcium imaging of AIB |
| pG-*npr-9p::mCherry* | *In vivo* calcium imaging of AIB (reference fluorescence) |
| pG-*lin-44p::GFP* | Marker |
| pG-*lin-44p::mCherry* | Marker |
| pG-*myo-3p::venus* | Marker |
| ApE files including detailed construction information of listed plasmids can be found in Supplementary file 6. | |
